# Collaborative work processes in establishing a MiniMaria treatment center for youth substance addiction: a qualitative inquiry of county council healthcare and municipal efforts

**DOI:** 10.1186/s12913-024-11820-4

**Published:** 2024-10-30

**Authors:** Maria Lindberg, Jofen Kihlström, Irene Hylander, Martin Salzmann-Erikson

**Affiliations:** 1 Department of Caring Science, Faculty of Health and Occupational Studies, University of Gävle, Gävle, 801 76 Sweden; 2 Centre for Research and Development, Region Gävleborg, Gävle, 801 87 Sweden

**Keywords:** Inter-organizational collaboration, Communicative constitution of organizations, Four flows model, Social work, Health care, Organizational studies, Organizational behavior, Adolescent health services, Addiction medicine, Group processes

## Abstract

**Background:**

This article is part of a larger study exploring the collaborative dynamics between key stakeholders in providing care to youths suffering from alcohol or substance use and their families in formulating policies and operational practices for county and country-wide application in similar settings. The focus of this article is to describe the collaborative processes between two stakeholders, a municipality, and a county council, in establishing a MiniMaria treatment center. While collaborative efforts between municipalities and county councils in health service provision are often acknowledged, little is known about how communication and decision-making processes between these entities shape the success of such initiatives. This study aims to fill this gap by providing insights into the communicative processes that foster organizational cohesion, agility, and innovation. The guiding research question is: What communicative processes occur between the county council and municipal stakeholders during the planning phase of the MiniMaria treatment center?

**Methods:**

The municipality and county council were selected based on purposive sampling, owing to the proximity and accessibility of the field. An exploratory and descriptive design, incorporating a participatory research approach, was employed for this qualitative investigation.

**Results:**

Two central themes, each underpinned by specific subthemes sum up the essence of our findings. The first theme underscores the collaborative dynamics and shared objectives that have emerged through the project, thus showing the importance of a unified vision and mutual understanding in driving the initiative forward. The second theme points to the practical aspects of implementing the project, including recruitment strategies, and the significance of interpersonal communication.

**Conclusions:**

This article sheds light on the establishment of a MiniMaria treatment center through collaboration between a municipality and county council, using the Four Flows Model to interpret communicative processes. Membership negotiation was crucial for defining roles and building a unified team identity, while activity coordination ensured aligned stakeholder efforts. Self-structuring facilitated internal organization and operational clarity, and institutional positioning aligned the initiative with broader healthcare norms, enhancing its credibility and impact. These communicative practices were central to get a grip on inter-organizational complexities, emphasizing communication’s constitutive role in organizational development and innovation.

**Supplementary Information:**

The online version contains supplementary material available at 10.1186/s12913-024-11820-4.

## Background

Addiction and substance use among adolescents is a major problem in Europe and Sweden, and it has been the topic of intensive study in recent years [1–4]. In Europe, one of the most serious public health issues is the use of psychoactive drugs among young people, which has been related to a variety of negative effects such as poor physical and mental health, poor academic success, and an increase of risk-taking behaviors [1, 3–5]. In Sweden, the prevalence of alcohol consumption, tobacco use, and engagement with illicit substances among youth is notably high, constituting a principal concern for health care practitioners and policy makers alike [4, 5]. The ramifications of substance consumption on the mental health of young individuals are profoundly troubling. Empirical evidence underscores a robust correlation between substance consumption and the onset of mental health disorders, such as depression, anxiety, and suicidal tendencies as well as what is referred to as activity addictions [6–8]. The idea of activity addictions has been contested but has gained more acceptance in recent research [9, 10]. Empirical evidence also shows a high comorbidity, up to 54% in a group receiving substance use treatment in a study, between substance use and gaming addiction. Research also point to the conclusion that different behavioral addictions such as gaming, gambling, sex addiction and shopping can be exacerbated when abstaining from psychoactive substances [11–13].

Research findings highlight the significance of integrated treatment centers that provide a combination of behavioral and family therapies within a single facility. This approach proves more beneficial than treatment from mental health services alone, which did not show as marked an improvement for youth with dual diagnoses. Therefore, integrated treatments that simultaneously address substance use disorders and psychiatric conditions are regarded as the optimal standard [14, 15]. Nonetheless, the success of integrated care is also dependent on the teamwork and methodology employed by the staff. Lowis et al. [16] identified challenges to implementing evidence-based interventions in integrated settings, noting that factors such as staff competence, policies, programs, routines, and the overall organization can significantly influence the success of the integrated unit.

In a study, Ekendahl [17] embarked on an exploration of the methodologies and philosophical underpinnings guiding the operations at MiniMaria treatment centers, also called MiniMaria, designed to support young individuals grappling with substance use challenges, notably cannabis, in Sweden. This investigation, through qualitative interviews with the center’s staff, sought to unveil the strategies employed to address youth substance use complexities. Moreover, it highlighted MiniMaria’s dedication to crafting individualized care plans, underscoring the imperative of adapting interventions to the unique profiles and predicaments of young clientele, thus championing a versatile and reactive treatment paradigm within the confines of prevailing structural norms. Evidence-based interventions that target individual and environmental risk factors have been shown to be effective in reducing substance use among young people [3, 15]. The resulting conclusion suggests that MiniMaria’s approach exemplifies a seamless integration of evidence-based practices with personalized care, representing a comprehensive strategy in addressing substance use among young people.

The focus of integrated care instead of cooperation around young people with co-occurring mental health and social problems comes mainly from two forms of obstacles that professionals meet when working with young people with a palette of problems. Firstly, there is a focus on the care takers need to get help with a life situation rather than getting specialized help from many caregivers simultaneously, and secondly the inefficient use of resources and inadequate systems for overcoming organizational and legislative obstacles [9, 13]. A Swedish study found that the Coordinated Individual Plans (CIP) model used in Sweden for cooperating and defining responsibilities between parties is inadequate and, in most cases, have a poor outcome. The stakeholders need for cooperation and defining responsibilities comes from that the County council have the responsibility for medical and psychiatric care in the county. The other stakeholders are the municipalities that have the responsibility for social services and social care, there are ten municipalities within the county. In total there are four stakeholders that need to cooperate as the project involves three municipalities within one county council.

It is imperative to find new ways to meet the needs of young people in socially vulnerable positions in a way that is beneficial for them as well as resource effective [18]. MiniMaria as a treatment center has the ambition to fill the gap between the health care system and social workers while at the same time embracing evidence-based treatment models and individually suited care plans. Despite the existing research on collaborative governance in public health, there is a lack of empirical studies focusing on how communicative practices between different governance structures, such as municipalities and county councils, influence the success of health services initiatives. This study addresses this gap by investigating the communicative and collaborative processes involved in establishing a MiniMaria. The findings contribute to our understanding of the constitutive role of communication in fostering cohesion, agility, and innovation within public organizations. Understanding the process of starting up a MiniMaria treatment center where both stake holders need to diverge from praxis and divisions of responsibility between their respective organizations will prove useful in similar endeavors in the future.

### Classifications of diagnoses

The International Classification of Diseases (ICD) and the Diagnostic and Statistical Manual of Mental Disorders (DSM) are two leading classification systems for mental disorders, each developed under different auspices and for varying purposes. The DSM underscores cultural nuances in psychiatric diagnosis facilitating an in-depth understanding of mental disorders across diverse cultural landscapes. In contrast, the ICD has broadened its scope by recognizing behavioral addictions such as ‘Gaming Disorder,’ reflecting a more inclusive view of addiction beyond substance use. The DSM primarily categorizes traditional substance use disorders under ‘Substance-Related and Addictive Disorders’. Substance use and activity addiction, which is the focus of MiniMaria, is defined as a pattern of repeated and harmful behavior that is driven by an intense craving or urge, often despite negative consequences. Hence, addictive behavior at MiniMaria will include youths demonstrating harmful or risk-taking behavior towards gambling, sex, shopping, eating, and even technology use [9, 10].

### Theoretical framework

Ontologically, we perceive organizations not merely as static entities, but as dynamic constructs continually shaped through communicative processes. This perspective aligns with the framework of Communicative Constitution of Organizations (CCO) [19]. CCO is a contemporary approach that emphasizes the critical role of communication in shaping organizational structures, processes, and identities. This perspective asserts that organizations are dynamic and constantly evolving through the communicative practices of their members. Epistemologically, this theoretical approach was deemed particularly appropriate for exploring the establishment of the MiniMaria treatment center, where multiple stakeholders negotiate, coordinate, and restructure their collaborative efforts through intricate communicative interactions. A key contribution to CCO is the “Four Flows Model”, which outlines four vital communication flows: membership negotiation, activity coordination, self-structuring, and institutional positioning. These flows work together to establish and maintain an organization’s identity, roles, and relationships, both within the organization and externally. The CCO approach has become increasingly popular in organizational communication studies, as it underscores the dynamic nature of organizations and highlights the significance of communication processes in understanding organizational life. For example, a Finnish study by Levonius and Sivunen [20] explored the role of social media in shaping the daily practices of care workers within an elder care organization. Employing the Four Flows Model, the researchers illustrated how care workers used social media platforms to navigate and redefine their roles and the organizational structure. The Four Flows Model, provided a lens through which the interplay between care workers’ agency and the organizational structure was examined. The study highlighted the dynamic nature of organizational communication mediated through digital platforms, demonstrating how these interactions contributed to the ongoing constitution of organizational identities and practices. By considering communication as the foundation of organizational existence, CCO offers a fresh outlook on organizational studies, opening new research possibilities and practical applications. While the Four Flows Model did not dictate the initial stages of our data collection or analysis, it was crucial in the later stages of interpreting the complex layers of data collected, framing our discussions, and interpreting of the collaborative dynamics observed. The framework provided a conceptual scaffold that highlighted the centrality of communication in mediating and shaping organizational structures and identities, enriching our understanding of the collaborative efforts within the MiniMaria initiative.

## Methods and material

### Aim

This article is part of a larger project exploring the collaborative dynamics between key stakeholders in providing care to youths and their families. It touches upon partnership elements and quality improvement’s role in fostering effective, person-centered care, while also reflecting on the experiences of treated youths and their families for a full view of MiniMaria’s services. The goal extends to formulating policies and operational practices for county and country-wide application. The primary aim of this article was to describe the collaborative processes between two stakeholders, a municipality, and a county council, in establishing a MiniMaria treatment center. The guiding research question is: What communicative processes occur during the planning phase of MiniMaria between the county council and municipal stakeholders?

### Design and setting

An exploratory and descriptive design, incorporating a participatory research approach with the focus on a joint process of knowledge-production to get to new insights on the part of both researchers and the participants, was employed for this qualitative investigation. The municipality and county council were selected based on purposive sampling, owing to the proximity and accessibility of the field. The project leader for establishing the MiniMaria treatment center initiated contact with the university, requesting collaboration to follow the processes. The project leader served as both a gatekeeper, facilitator for the researcher’s entry into the field, and a research subject.

### Participants

Before our entry into the field as researchers, two primary groups had been established: a steering group and a project group. The steering group comprised six administrative staff in chief positions, representing both the county council and the Municipality. The project group consisted of 13 individuals, primarily occupying first and middle-line managerial positions from both organizations. Additionally, a third group, the management team, existed, consisting of three members with the primary responsibility of hiring staff to the new unit. Two of these members were part of the project group.

### Data collection

All four researchers were engaged in data collection by observing the steering and project groups’ meetings. Although not all researchers participated in the same specific meetings, at least two researchers attended each one. The steering group were responsible for making strategical decisions for the project and the project group were responsible for discussing and making operating decisions regarding MiniMaria. In total, we attended six meetings with the steering group and five meetings with the project group. At all meetings researchers took extensive notes, and one meeting was also audio recorded. After each meeting, the researchers convened and discussed the meeting: what were our overall impressions, what topics were discussed, and what decisions were made? How did the attendees address each other, and what processes were evident? We wrote analytic notes based on our joint discussions of what we observed and our interpretation of the discussions. After some of the initial meetings (in total two steering group meetings and three project group meetings), IH contacted one or two participants and asked for a follow-up interview. These interviews were semi-structured and focused on the attendees’ experiences during the meeting. The interview guide is presented in Supplementary Material (A) The interviews were audio recorded and conducted either by phone or via Zoom. Immediately after each interview, IH wrote a 1–2-page summary. The audio files were stored on the university’s cloud servers, allowing all researchers to listen to the interviews in full length (in total six interviews, range 14–34 min, mean 16 min). To further understand the group processes, IH conducted retrospective semi-structured interviews. The questions in these interviews addressed the individual perspective and perceptions of the MiniMaria project, for example: How were you assigned to the project? What expectations did you have from the beginning? What challenges have you encountered so far? The full semi-structured interview guide is presented in Supplementary Material (B) These retrospective and not time-sensitive interviews were held with all participants from both groups (*n* = 19). The interviews were mainly grounded in the interview guide, but as the researchers became more familiar with the processes and understanding of the group processes, the questions were slightly revised as more probing questions were asked and new questions arose based on insights from previous interviews.

### Data analysis

**Fig. 1:**
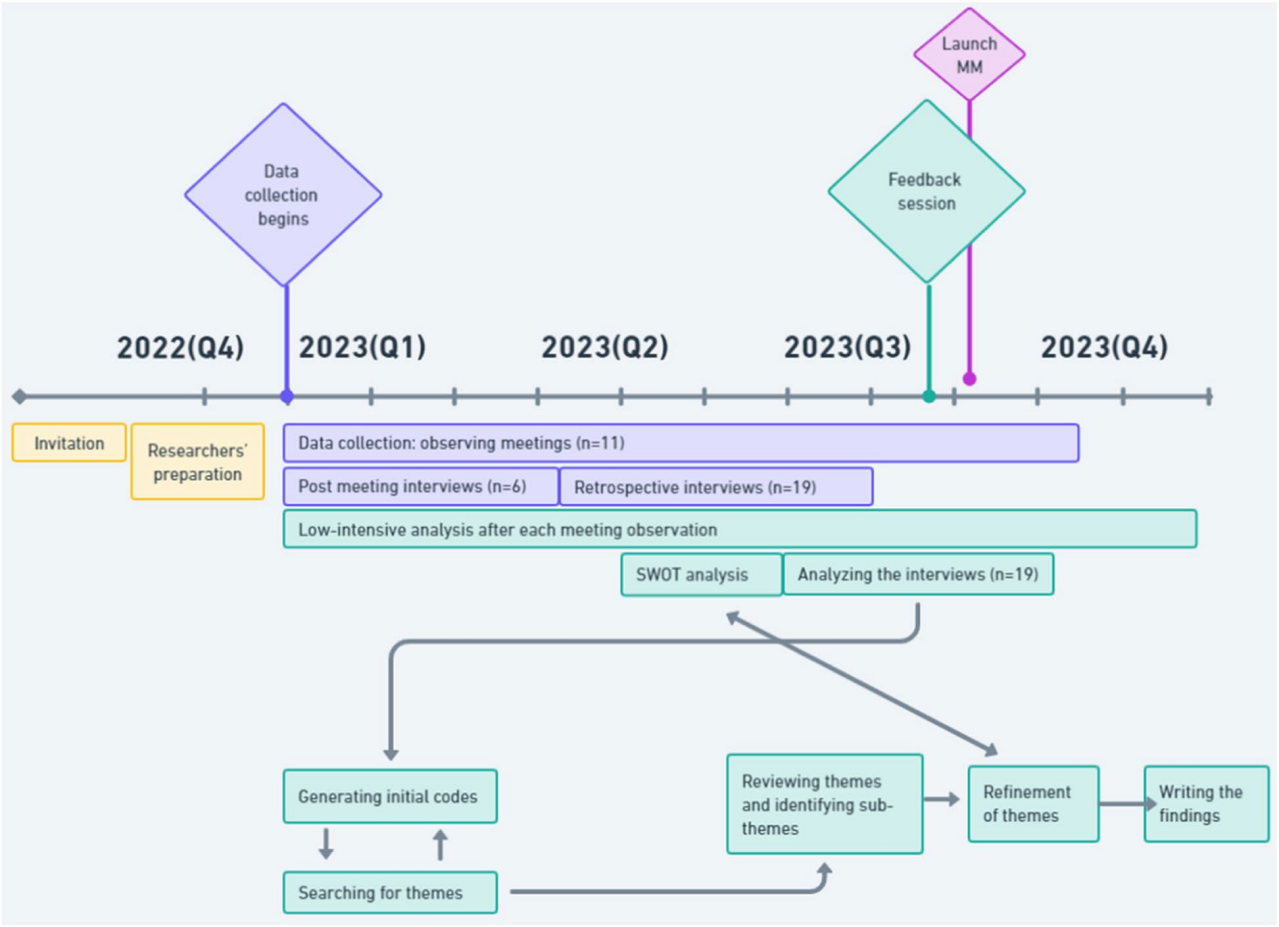
Overview of the data collection and data analysis

The data collection and data analysis took place in tandem, thus being a non-linear process. For an overview of the data collection and data analysis, see Fig. 1. In our post-meeting sessions, we interpreted the groups’ processes and wrote analytical memos. The transcribed summaries from the interviews were read through repeatedly and then inductively analyzed to identify what the participants had talked about, that generated initial codes. Based on similarities, the codes were sorted into content areas and in the next step into named themes and subthemes. We then conducted a deductive analysis based on strengths (*n* = 30), weaknesses (*n* = 40), opportunities (*n* = 20) and threats (*n* = 20) (SWOT-analysis) on the interpreted data from observations and interviews. Just before opening of the MM, we presented our initial analysis to the steering and project groups.

### Rigor

In qualitative research, ensuring the credibility of findings through methodological rigor is paramount. Our study employed several strategies aligned with established criteria [21]. One essential strategy was conducting respondent feedback sessions, where participants validated preliminary findings. This process, which included both virtual and in-person interactions, allowed participants to confirm that the findings accurately reflected their experiences, thus enhancing confidence in the truthfulness of the research outcomes. The study’s credibility was further reinforced by our use of prolonged engagement and persistent observation. The extended time spent in the field enabled the research team to develop a deep understanding of the context and phenomena under study. Transferability was enhanced through the provision of descriptions within our findings, offering a comprehensive portrayal of the context, for example, quotes; thereby enhancing the external validity of our research. Dependability and confirmability were demonstrated through the methodical approach of rotating researchers during data collection. This strategy ensured that diverse perspectives were incorporated, maintaining consistency in the findings across different observers and over time. Our iterative review and discussion of notes and interviews further strengthened dependability by ensuring that interpretations were consistent and accurately reflected a comprehensive understanding of the data.

## Results

The Results section is structured around two central themes, each underpinned by specific subthemes that encapsulate the essence of our findings. The first theme explores the collaborative dynamics and shared objectives that have emerged through the project, highlighting the importance of a unified vision and mutual understanding in driving the initiative forward. The second theme delves into the practical aspects of implementing the project, including recruitment strategies, the significance of interpersonal communication, and the establishment of effective networks with educational institutions and other relevant entities. For an overview of sub-themes and themes, see Table 1.

**Table 1:** Overview of sub-themes and themes

Sub-themes	Themes
Identify needs and relating to decision-making processes	The complexity of coordinating collaboration: driving forces and challenges in the planning of the MiniMaria treatment center
Awareness of the importance of integrating and collaborating	
Managing divergences, disagreements, and roles	
The sentiment of community bolstered by shared expectations and points of contact	Towards a unified vision and optimism for the future: unifying expectations and clarifications with a positive outlook on continued collaboration
Fostering collaborative synergy with mutual trust	

### Theme 1: The complexity of coordinating collaboration: driving forces and challenges in the planning of the MiniMaria treatment center

The theme is divided into three subthemes that detail MiniMaria’s foundation, the commencement of the project, and the obstacles faced. The project’s objective was to improve cooperation between the municipality and the county council regarding substance use among youth, aiming to pinpoint and address support deficiencies for young people by establishing an integrated treatment center. The genesis of the project was a drawn-out endeavor, championed by influential individuals, amidst diverse views on the necessity of the treatment center. Challenges in collaboration emerged due to legal inconsistencies, conflicting priorities, and a lack of adaptability. The county council’s hesitance was rooted in skepticism about the support requirement, citing a lack of comprehensive data. They stressed the importance of complying with existing laws, regulations, and care protocols, which limited the scope for flexibility.

### Identifying needs and relating to decision-making processes

The beginning of the collaboration was shaped by the urgent needs identified in the project group, highlighted by the challenges faced by young individuals. This initiative was shaped in response to a national comorbidity study, aiming to fill healthcare gaps.

“*We looked at how other municipalities had addressed the issue*,* focusing on our target group. We observed a high degree of comorbidity in our groups*,* where the youth did not receive the contact they needed.*” (Interview 9, project group member).

The urgency to identify county-wide issues was complicated by differing needs perceptions between collaborators. The need mapping process was demanding and requested significant time, as the process was complicated by the county councils’ skepticism about the project’s necessity and impact, while the municipality affirmed its importance. The planning of the MiniMaria was influenced by the growing normalization of substance use, the need for new intervention methods, and existing support system complexities. A clear mission statement was deemed essential in early planning, despite challenges in aiming for county-wide expansion. The mission statement emerged as a critical early planning element. A significant planning obstacle was the need for each participating organization to internally validate and process all decisions, leading to a perception of prolonged timelines for finalizing decisions.

“*We had different views on needs and working methods from the municipality and the county council. We were far apart for a long time. Then*,* when a decision was finally made*,* we could still agree on something.*” (Interview 7, steering group member).

Support from county council leadership was seen as vital. The lack of clear decision-making protocols was pinpointed as a key reason for delays. To strengthen the decision-making process, a steering group was formed with representation from both stakeholders. The steering group was responsible for making initial decisions about management involvement in the project group and guaranteeing the project’s ongoing progress. Participants stressed the critical need for a clear mission statement yet faced hurdles in spreading this approach county-wide. The mission statement was seen as crucial in the early planning stages. However, there were concerns about the project leader’s role and the project plan’s initial structure. Participants called for clear quality indicators for future evaluations, noting their absence as a significant gap. Challenges included navigating the complexities of addiction care interfaces, leading to debates over responsibilities and fears that existing operations could overlap with the new initiative. Many participants, with a strong focus on youth issues in municipal services, brought a long-standing interest in this area, greatly influencing the project’s start.

*What the purpose of MiniMaria is. The target group one envisages*,* your friend*,* your parent who is worried. It’s supposed to be before the addiction clinic is the idea. How to make it known to the public so they know that this is where to turn with these problems? The stakeholder analysis we conducted revealed stakeholders I didn’t know existed*,* especially in the municipal part. How do you ensure that people turn to MiniMaria? How do we make this known with MiniMaria that it should be out to the public? There*,* I have reflections and see challenges. How does one trust this?* (Interview 3, project group member)

### Awareness of the importance of integrating and collaborating

Participants described the field of substance use treatment as particularly challenging, highlighting the need for careful planning to ensure the new initiative’s effectiveness. Although opinions varied on the necessity of the initiative, there was agreement that no single entity should shoulder the responsibility for establishing the required functions alone.

“*We realized that we cannot work with the target group alone as social services; we need to involve the county council since mental health is their area of responsibility and it is commonly encountered.*” (Interview 4, project group member).

Participants noted the challenges of aligning different legal frameworks and the lack of understanding between organizations’ operational cultures. Concerns about legal compliance were especially significant for shared documentation, with a collective aim to avoid errors while capturing complete information. While strict adherence to laws was seen as crucial for accuracy, too much focus on details was sometimes seen as a hindrance. There was a clear preference for a centralized administrative approach and the integration of efforts across multiple municipalities. Past difficulties in inter-organizational collaboration were seen as possible barriers to the current project, especially with many other initiatives running simultaneously. The level of commitment from both entities was uncertain. Building engagement, understanding each other’s operations, and drawing on past successes were deemed essential for overcoming potential challenges in collaboration. The ethical commitment to addressing substance use was universally recognized, with each participant playing a unique and vital role in the project. The strategy also highlighted the importance of involving more partners, encouraged by directives from county leadership.

“*I believe that this is also a process within the project group that needs to be discussed and it’s good that it’s being addressed. We achieve a mutual understanding and a preconception since we have different fundamental missions*,* but we are now going to work with a common target group. The team [author’s note: the participants refer to the staff at the treatment center] should contribute from their perspectives*,* based on their initial assignments. That’s the goal.*” (Interview 17, steering group member).

The early inclusion of additional partners, facilitated by the municipality and county council leadership, was stressed, exemplified by the Drug-Free School initiative in primary and secondary education, aligning with the project’s goals. Utilizing established collaborative networks was deemed significant. The steering group composition, including county council leadership, social service leadership, a school principal, and particularly the operations manager, was considered crucial due to the impact on departmental functions. However, the roles between the steering and project groups were seen as vague, with some participants feeling they joined the project group later than ideal.

### Managing divergences, disagreements, and roles

Participants described the project as lengthy and challenging, hindered by less-than-ideal circumstances. Resource constraints were noted not just as obstacles to productive collaboration, but also as barriers to tackling the intricate social challenges faced by the project’s target group. The issue of location significantly delayed efforts, contributing to a sense of disarray amidst the numerous stakeholders. An imbalance in influence was evident, with the county council’s dominance making it difficult for the municipality to have its perspectives adequately recognized. The county council was perceived as operationally knowledgeable yet inflexible regarding task allocation, whereas the municipally was viewed as efficient and adaptable but not deeply rooted in evidence-based practices.

” *The local issue was a critical point last winter. It took time to come forward and I tried to push our technical office to find solutions. No direct assignment I took on*” (Interview 7, steering group member).

The project group engaged in numerous discussions, which occasionally created a sense of stagnation, and the steering group’s rejection of their proposals as unsuitable or unnecessary contributed to dwindling patience. However, diverse perspectives were seen as conducive to productive discussions and solutions. Friction was expected as a precursor to alignment, with a growing consensus on MiniMaria’s target youth demographic emerging through the process. Communication about and integration of the Drug-Free School initiative into the project, was considered ambiguous. The participants noted the absence of school involvement in the process and the challenge of allocating time for discussing the Drug-Free School initiative in project meetings. Additional hurdles encompassed local conditions, varying expectations, and feelings of exclusion among smaller municipalities due to inconsistent representation in project meetings. The issue of competence needed at the treatment center emerged as contentious, particularly regarding the perceived necessity for registered nurses. Concerns were raised that staff might hesitate to fully utilize their skills fearing that others in the interdisciplinary team were more expert, which could impede day-to-day operations.

The project leader was seen as the connector between the steering and project groups. However, there were uncertainties regarding roles, with key individuals perceived as not being sufficiently involved. Participants reported considerable challenges in scheduling meetings and difficulties in prioritizing attendance, despite a keen interest in participation. Physical meetings fostered a sense of shared purpose, but the absence of a common meeting place, due to differing organizational affiliations, posed a challenge. Digital meetings, on the other hand, were reported to function effectively. Some participants stressed the importance of actively contributing to the work to dispel any notions of non-participation. Arriving unprepared for meetings was viewed as an obstacle to progress, and asking critical questions was deemed essential for success. A lack of knowledge was identified as a factor that could misdirect the focus of the work.

“*A lot of physical meetings at the start were necessary. Wouldn’t have gotten through with just digital meetings*,* you have to meet in person. Something happens in the meeting when people meet face-to-face*,* pushing forward*,* increasing personal contacts. Helping to ‘like each other*’ *a bit.*” (Interview 4, project group member).

### Theme 2 - Toward a unified vision and optimism for the future: unifying expectations and clarifications with a positive outlook on continued collaboration

The theme encompasses two subthemes that trace the journey towards a unified vision, despite initial challenges. Over time, uncertainties diminished, and the work gained clarity and focus. The critical role of involving diverse stakeholders, such as the Drug-Free School initiative, was accentuated. The decision to initiate the MiniMaria treatment center helped align various components, breeding optimism about the necessary elements for success. Personal attributes and historical relationships were identified as enablers of cooperation, with consistency, engagement, and a commitment to youth welfare highlighted as key to achievement. Additionally, the value of thorough evaluation and consistent follow-up was emphasized.

### The sentiment of community bolstered by shared expectations and points of contact

The participants attributed the project’s strength to the expertise within the group, leading to well-defined objectives and alleviating concerns, thereby fostering a collaborative atmosphere with a unified vision and shared accountability. The dynamics of operating within a politically governed framework were seen as both a driver of progress and a source of cautious optimism. The venture was deemed exciting but securing the appropriate expertise for the treatment center identified as an ongoing challenge. There was consensus on the necessity for specialized knowledge and skills among treatment center staff.

“*I think that basically it is the clear direction of will from our politicians that we should have this. Then everything else becomes a non-issue. The merit of working in a politically controlled organization*” (Interview 15, steering group member).

The synergy from combined expertise and coordinated actions was considered a key asset for MiniMaria, which aims not just to address substance use but also to deter involvement in criminal activities, build consensus, and facilitate family-wide collaboration. The treatment center’s strategy involves early, preventive interventions in families, with an eye on long-term outcomes. The Drug-Free School initiative is expected to engage many young individuals, who would then be seamlessly integrated into MiniMaria’s services. Thus, all partners, including smaller municipalities, were seen as crucial to the overarching goal of supporting youth, highlighting their role in this significant endeavor. The youth clinic was also mentioned as an important partner in collaboration. The process has revealed opportunities to bridge gaps and combine efforts from various sectors, underscoring the ongoing need to seek out points of connection. The participants hold high expectations for the forthcoming work and highlight the significance of collective recognition of the project’s value. A sense of optimism has taken root among the steering group and project group, cultivated through shared learning, joint problem-solving, and respectful discussions. The efforts are grounded on a strong foundation, albeit with an acknowledgment that not all aspects will proceed flawlessly. Concerns were voiced regarding potential disagreements among participants in the future and their implications for the project’s progress.

*“We need to support the patient for a bit longer and perhaps enter each other’s spaces and meet on the ‘bridge’ to help one another.”* (Interview 5, project group member).

### Fostering collaborative synergy with mutual trust

Dedicated and involved individuals were seen as beneficial to the collaborative effort, enhancing autonomy, and ensuring the participation of all involved. As the project progressed, elements began to coalesce, with a shared sentiment that collective contributions led to greater achievements. The participants noted a dissipation of earlier tensions, now replaced by a positive outlook and confidence in the ongoing process. They recognized that the project and accompanying changes had consumed considerable time, a common occurrence in such endeavors. After sustained involvement and dialogue between steering and project group members, a consensus emerged, bringing clarity to the proceedings. There was a palpable sense of momentum in the practical work. A strong sense of duty, drive, and commitment was reported, with a focus on promoting health, preventive measures, early intervention, and the spread of knowledge. Visiting other MiniMaria sites was described as a practice that spurred progress. The participants voiced a desire for more opportunities to pose questions in the future. Personal interactions were deemed helpful in overcoming challenges. Taking time for thoughtful discussion and mutual reflection within the project was highlighted as crucial, along with the ability to fine-tune the pace of work as needed. Anticipation for the opening of the MiniMaria treatment center was high, despite remaining uncertainties that needed resolution. In achieving effective collaboration, fostering a collective “we” mentality was deemed crucial, alongside the need for leadership and drive within the group itself.

“*Thanks to our collective process*,* we have now jointly arrived at a clear purpose and objective. That one adheres to our collaboration agreement*,* engages*,* is honest*,* raises difficult questions but also to be secure in the structure are of importance. The staff should know where to raise their questions. That the steering group continues in the same good spirit and that we raise questions and can provide feedback on them.*” (Interview 17, steering group member).

Key elements for the project included transparency, consistency, active involvement, adaptability, and preserving the harmonious atmosphere that had been established. Participants felt supported by their organizations in developing roles and methodologies collaboratively, leading to a shared understanding of the treatment center’s operation and the execution of school outreach in partnership with the Drug-Free School initiative. Significant progress was marked by completing staff recruitment and modifying facilities. Ensuring the initiative’s credibility and expanding networks were highlighted as vital for continued progress. Confidence in the project group was strong, though one member critiqued their own preparation. There was a consensus on maintaining momentum and achieving objectives despite organizational rigidity, stressing perseverance in the face of obstacles and the importance of reciprocal support. The intention is for MiniMaria to exemplify success, underscoring the necessity for ongoing evaluation of the treatment center’s work.

“*Then*,* once in place*,* it’s important to show that MiniMaria exists*,* through marketing*,* our mission*,* target group*,* and by building our contacts with schools and other arenas and operations that meet this target group.*” (Interview 4, project group member).

## Discussion

The primary aim of this article was to describe the collaborative processes between two stakeholders, a municipality and a county council, in establishing a MiniMaria treatment center. The central research question regarded the processes that unfold during the planning stage among stakeholders from county council and municipal levels in the steering group and project group. Our findings reveal a multifaceted landscape of collaboration between municipal and county council stakeholders. The first theme highlights the intricate dynamics of cooperation, underscored by a shared commitment to a unified vision, which proved essential in overcoming institutional barriers and aligning diverse organizational cultures and priorities. This theme emphasized the importance of a cohesive strategy, facilitated by intensive negotiations and mutual understanding. The second theme delved into the practicalities of implementing the project, focusing on the significance of interpersonal communication, effective recruitment strategies, and the establishment of robust networks with educational institutions and other relevant entities. These practical aspects were critical in maintaining momentum and ensuring the project’s success, despite the challenges posed by legal constraints and differing operational protocols. Together, these themes illustrate the complexity and dynamism of inter-organizational collaboration in innovating adolescent care services through the MiniMaria initiative. As previously noted, our study fills a critical gap in the existing literature by offering detailed insights into how communication functions as a constitutive element in fostering organizational cohesion and innovation. This contribution extends the existing understanding of organizational communication within public sector collaborations, particularly in health service provision. In the forthcoming part, we discuss how these findings relate to the CCO framework.

The CCO framework [19] posits that organizations are not merely entities using communication as a tool but are fundamentally constituted through communicative practices. Our empirical insights reveal that communication acts, not merely as a conduit for information or a mechanism for coordination, but as the foundational process through which organizational meanings are created, contested, and transformed. This perspective brings to light the dynamic interplay between communicative practices and organizational structures, where each is continually shaped and reshaped through the other. Such a view underscores the fluidity of organizational boundaries, identities, and hierarchies, highlighting the role of communication in facilitating organizational adaptability and change. The findings from our study demonstrate how communicative practices are pivotal in navigating the complexities when an integrated organization is established from an inter-organizational collaboration. This includes the formation of organizational identities, the enactment of roles and relationships, and the engagement with the wider institutional landscape. Through this lens, our study offers nuanced insights into the constitutive power of communication in organizations, emphasizing its centrality in fostering organizational cohesion, agility, and innovation. In the subsequent discussion, we will interpret our findings in relation to the key concepts of the Four Flows Model, namely, membership negotiation, activity coordination, self-structuring, and institutional positioning.

### Membership negotiating

The negotiation phase within the MiniMaria initiative, as delineated through the CCO framework, represents a critical juncture in the project’s inception and progression [19]. This phase is characterized by the genesis of the project through a series of planning sessions, where the communicative process played a pivotal role in shaping the collaborative efforts of both the steering and project groups. For instance, the process of mapping out needs required significant time due to differing perspectives on the initiative’s importance and potential impact. One party’s skepticism contrasted with the other’s consistent recognition of the pressing need, highlighting the essential role of communicative negotiation in reconciling these differences and aligning the stakeholders’ objectives. Participants in the study described the project as “lengthy and challenging,” with resource constraints noted as obstacles to productive collaboration. These challenges underscored the importance of the ongoing communicative process in managing the different organizational cultures and operational approaches of the partners involved.

The essence of the CCO framework, which posits that the very fabric of organizations is woven through the continuous interplay of interactions and discourses among members, finds a concrete manifestation in our study. We observed that the negotiation phase was not merely about logistical arrangements but evolved into a critical process of collective identity construction. This was key in overcoming the challenges of working across different organizations. It highlighted the essential role of communication, not just as a minor activity, but as the central element of how organizations operate, grow, and develop. In the context of the MiniMaria initiative, the intricate tapestry of membership negotiation emerged as a foundational element. Stakeholders from both county council and municipal levels engaged in detailed discussions to delineate their roles, responsibilities, and contributions, demonstrating the vital role of communicative practices in defining and evolving organizational memberships. As the project progressed, the initial disagreements and differing perspectives gradually gave way to a more unified vision, illustrating how communication served as the vehicle for aligning diverse organizational cultures. This iterative process of dialogue and adaptation not only solidified roles and responsibilities but also allowed the organization to refine its mission and operational strategies in response to emerging challenges. Over time, these communicative exchanges not only established a cohesive organizational identity but also enabled the MiniMaria initiative to adapt to shifting external demands and internal complexities, demonstrating the fluid and evolving nature of organizational identity formation.

Furthermore, Baldwin’s and Magnusson’s [22] qualitative interpretivist study on program planner dignity and negotiation in collaborative projects offers valuable insights into the dynamics of negotiation within collaborative partnerships. The emphasis on practice-focused relationships and the impact of organizational hierarchy on planner dignity resonate with our observations on the negotiation phase within the MiniMaria initiative. Baldwin and Magnusson [22]underscored the importance of fostering interactions that affirm dignity and navigating the constraints posed by organizational hierarchies, paralleling our discussion on the dual role of the project leader and the critical need for maintaining stakeholder trust. Brattström et al. [23] extend this discourse by illustrating how initial trust perceptions, shaped by past successful collaborations and reputational strengths, can evolve through complex negotiations, impacting the collaborative dynamic. The perception of bias, or even its mere possibility, could undermine stakeholder trust. Thus, it is crucial to understand that trust, akin to a currency in inter-organizational cooperation, must be continually earned and upheld by all participating entities. This ongoing maintenance of trust is fundamental to fostering a collaborative and integrated identity essential for effective teamwork and collective action in multifaceted stakeholder environments.

### Activity coordination

Activity coordination within the MiniMaria project is not merely a logistical challenge; it represents a complex communicative act that bridges diverse professional landscapes, aligning the efforts of stakeholders from various disciplines. The coordination process involves a meticulous balancing act, where the timing, resources, and objectives of different activities must be harmonized to create a cohesive operational rhythm [24]. This harmonization is essential for maintaining the momentum of the project, ensuring that all stakeholders are engaged and that their contributions are effectively integrated into the initiative’s workflow. Our results highlight several instances of these coordination challenges. Participants reported considerable difficulties in scheduling meetings and prioritizing attendance, which underscores the importance of effective communication strategies to manage such logistical hurdles. Additionally, the need for each organization to internally validate and process all decisions further complicated the coordination efforts, leading to perceived delays in finalizing decisions. These challenges illustrate the multifaceted nature of activity coordination in the MiniMaria initiative. Moe et al. [25] underscore the benefits of informal, spontaneous interactions, highlighting their role in enhancing information sharing, problem-solving, and decision-making within teams. In contrast, our study reveals the challenges faced by the MiniMaria team in scheduling meetings and managing team collaboration, thereby identifying a significant gap in leveraging the potential of unplanned meetings. Despite encountering difficulties in aligning schedules and facilitating effective communication, the insights from Moe et al. [25] suggest that embracing the spontaneity of unplanned interactions could significantly improve the MiniMaria project management strategies and team dynamics. These challenges are not insurmountable; rather, they underscore the need for robust communication strategies and flexible planning mechanisms that can adapt to the evolving needs of the project. These micro socio-psychological processes are pivotal in fostering an environment where stakeholders are not only engaged but also deeply committed, enhancing the project’s adaptability and performance through inter-organizational learning agility. This approach aligns with the need for robust communication strategies and flexible planning mechanisms [24]. Moreover, our findings underscore the importance of a shared understanding and mutual respect among stakeholders. Effective activity coordination is predicated on the recognition of each participant’s expertise and the value of their contribution to the project. We argue that this collaborative ethos fosters a sense of ownership and accountability among team members, driving the collective effort towards the realization of the MiniMaria initiative’s goals.

### Self-structuring

Transitioning from the negotiation aspect, which highlighted the pivotal role of communicative engagement in forging a cohesive team within the MiniMaria initiative, we now direct our focus towards the self-structuring flow [19]. This shift underscores the necessity of internal organizational structuring in facilitating the project’s operationalization and sustainability. The self-structuring flow is characterized by the development of internal rules, roles, and norms that guide interactions and decision-making processes among stakeholders. This flow is instrumental in creating a structured environment where responsibilities are clearly delineated, and operational protocols are established, ensuring the efficient progression of the initiative. The MiniMaria project, through its detailed project plan, exemplifies the essence of self-structuring. The project plan serves not only as a textual anchor that provides stability and clarity but also as a dynamic entity subject to reinterpretation and adaptation through ongoing dialogue among project participants. The project leader’s role in connecting the steering and project groups was crucial in defining internal roles and responsibilities, a key aspect highlighted in the results. The need for clear quality indicators and the challenge of aligning decision-making across organizations further illustrate the importance of self-structuring in ensuring effective implementation and accountability within the initiative. Hence, the project plan not only broadens our comprehension of policy documents as institutional artifacts, which Sedlačko [26] views as mere inscriptions that establish order, but elevates this understanding to a more dynamic realm. Drawing upon Smith and Turner’s [27] notion, we see the project plan as “Texts-in-Action” (p. 8), a concept that transcends static order to activate and mobilize organizational members. This interpretation suggests that the project plan, far from being just a document, acts as a catalyst that propels the strategic direction of the initiative. Thus, the project plan is not only a textual anchor providing clarity and stability but also a living document that evolves through continuous dialogue among stakeholders, reinforcing its role in the self-structuring flow of the CCO framework [19].

Building on this foundation, we incorporate insights from the Berta et al. [28] study, which emphasizes facilitation within healthcare as a guided interactional process that enhances the uptake and application of scientific knowledge. This facilitation process, akin to the self-structuring flow within the MiniMaria initiative, involves guiding interactional processes that enhance organizational learning capacity. This parallel suggests a complementary relationship between facilitation and self-structuring in fostering effective knowledge implementation within healthcare organizations. Further, the study by Lewin et al. [29] refines our understanding of absorptive capacity, highlighting the significance of internal and external routines in knowledge creation, transformation, and assimilation. This concept resonates with the self-structuring mechanisms within the MiniMaria initiative, as evidenced by the collaborative dynamics and practical aspects of implementing the project, including the importance of interpersonal communication and the establishment of effective networks, for example visiting other MiniMaria treatment centers. The initiative’s approach to identifying needs and relating to decision-making processes, as reflected in the planning phase, underscores the internal and external routines essential for knowledge creation, transformation, and assimilation. The urgency to identify county-wide issues, complicated by differing needs perceptions between collaborators, and the demand for a clear mission statement in early planning, mirrors the structured internal processes within MiniMaria that facilitate the adaptation and assimilation of new knowledge, thereby enhancing the initiative’s innovative capacity and effectiveness.

### Institutional positioning

Institutional positioning, the last element within the Four Flow Model of the CCO framework [19], significantly influences the MiniMaria initiative’s alignment with broader healthcare norms and its advocacy for an innovative approach in healthcare and social services. This strategic positioning bolsters the initiative’s credibility, encourages collaborative efforts, and impacts policy and decision-making in adolescent care. Our findings shed light on the MiniMaria initiative’s interaction with the broader healthcare environment, showcasing how deliberate communicative actions ensure the initiative’s practices and outcomes are in sync with established healthcare standards, thus enhancing its credibility and facilitating its integration into existing healthcare frameworks, thereby optimizing its impact and scalability. For instance, the support from county council leadership was deemed crucial in aligning the initiative with existing healthcare protocols, demonstrating the need for clear decision-making processes and leadership involvement to ensure the project’s progress. Moreover, drawing attention to a Swedish study by Olsson et al. [30], which highlights a gap in understanding around standardized care plans and their basis in evidence-based knowledge, underscores the ongoing need for clarity and standardization in care processes. Despite the study’s age, its findings resonate with the Swedish National Board of Health and Welfare’s [31] report which emphasis on developing safe care and work processes, including standardized care processes, national guidelines, and guidance. These elements are crucial for creating reliable and safe systems, advocating for the development and implementation of knowledge support and guidance, especially in security-critical processes, and the gradual phasing out of outdated knowledge supports, guidelines, and procedures. The MiniMaria initiative further exemplified institutional positioning by involving additional partners early in the process, particularly through the integration of the Drug-Free School initiative, which aligns with broader public health goals and reinforces the project’s strategic alignment with existing frameworks. Echoing Ekendahl et al. [32], the MiniMaria treatment centers’ dedication to creating individualized care plans and a flexible treatment framework reflects a strategic alignment with broader structural norms and healthcare expectations, enhancing the initiative’s innovative profile within the professional community. Additionally, the Drug-Free School initiative and its commitment align with international research supporting drug-free zones to reduce drug-related crimes [33], further solidifying the initiative’s institutional positioning. Past challenges in inter-organizational collaboration were acknowledged, with an emphasis on building engagement and understanding to align with broader institutional goals, ensuring the initiative could overcome potential barriers and integrate effectively with ongoing efforts. The critical role of family support in prevention and treatment, as discussed by Elkington et al. [34] and Esteban et al. [35], aligns with the MiniMaria initiative’s strategies, highlighting the protective impact of parental involvement and family communication against substance use among youths. Institutional positioning is further reinforced by engaging with policy and decision-makers, enabling the MiniMaria initiative to influence the regulatory and funding frameworks essential for healthcare services. By fostering constructive dialogues with key stakeholders, the initiative seeks policy changes that support its operational model and objectives, characterized by strategic communication that bridges the initiative’s insights with broader policy goals, aiming to create an environment conducive to the adoption and expansion of the MiniMaria approach.

### Study limitations

While several strategies were implemented to ensure methodological rigor, certain limitations should be acknowledged. One notable limitation in dependability arose from the absence of electronic recordings during meetings, which could potentially lead to missed nuances in fast-paced discussions. Nonetheless, all one-on-one interviews were electronically recorded and transcribed verbatim. The study did not include external audits, which might have further enhanced confirmability by providing an independent evaluation of the research process and outcomes. This absence could be seen as a limitation in our methodological framework, as external audits offer an impartial perspective on the accuracy and neutrality of the findings.

## Conclusion

This article delineated the collaborative processes between a municipality and a county council in establishing a MiniMaria treatment center, emphasizing the pivotal role of communicative engagement. Through intensive negotiations, stakeholders from diverse organizational cultures and priorities aligned under a unified vision, overcoming institutional barriers. The practicalities of project implementation, including effective recruitment strategies and establishing robust networks, were critical in ensuring the initiative’s success. The CCO framework further illuminated how organizations are constituted through communicative practices, with our findings highlighting communication as the foundational process through which organizational meanings are created and transformed. This perspective underscores the fluidity of organizational boundaries, identities, and hierarchies, emphasizing communication’s role in fostering adaptability and change. Our study showcased the constitutive power of communication in navigating the complexities of establishing an integrated organization from an inter-organizational collaboration. This includes forming organizational identities, enacting roles and relationships, and engaging with the wider institutional landscape, providing nuanced insights into communication’s central role in fostering organizational cohesion, agility, and innovation.

### Disclosure of use of large language model

The authors utilized OpenAI’s ChatGPT-4o for grammatical corrections and to improve the flow of the text, under our supervision, ensuring the academic content’s integrity. This was a supplementary step to enhance readability, given the authors’ diverse linguistic backgrounds. The use of ChatGPT did not influence the research findings or their interpretation.

## Supplementary Information

Supplementary Material 1.

Supplementary Material 2.

## Data Availability

The datasets generated and analyzed during the current study are not publicly available due to privacy or ethical restrictions but are available from the corresponding author on reasonable request. A data management plan was registered in DMPonline (reg. no 123662).

## Abbreviations

CCO: Communicative Constitution of Organizations
CIP: Coordinated Individual Plans
DSM: The Diagnostic and Statistical Manual of Mental Disorders
ICD: The International Classification of Diseases
