# Supplementary material for: Collaborative work processes in establishing a MiniMaria treatment center for youth substance addiction: a qualitative inquiry of county council healthcare and municipal efforts
Source: BMC Health Serv Res. 2024 Oct 30;24:1307. doi: 10.1186/s12913-024-11820-4 (PMC11523651; doi:10.1186/s12913-024-11820-4)
Supplement: Supplementary file 1 — Supplementary Material 1. [file 12913_2024_11820_MOESM1_ESM.docx]

**Interview guide after the meetings**

Yesterday, there was a meeting with the X-group that you attended. In this interview, I'd like to ask you some questions about the meeting.

- Tell me about any specific event or discussion during the meeting that you found interesting or significant.
  - Why was it important to you?
- What were your thoughts and feelings before the meeting, and how have they changed afterward?
  - Can you describe some of these changes?
- How would you describe the group's culture and atmosphere during the meeting?
  - How did it affect you and your participation?
- Is there anything you wish had been discussed in more detail during the meeting?
  - Why do you think that topic is important?
- Can you describe a situation from the meeting where you learned something new or gained a different perspective on something?
- How do you feel that your own experiences and skills contribute to the project and the collaboration between groups?
- If you could change something about the meeting's structure or how it was carried out, what would it be and why?
- What are your expectations for future meetings and collaboration in the project?
  - How do you think the group can work towards achieving these goals?
- How do you view the project's long-term potential, and what do you think will be crucial factors for success?
