# Supplementary material for: Collaborative work processes in establishing a MiniMaria treatment center for youth substance addiction: a qualitative inquiry of county council healthcare and municipal efforts
Source: BMC Health Serv Res. 2024 Oct 30;24:1307. doi: 10.1186/s12913-024-11820-4 (PMC11523651; doi:10.1186/s12913-024-11820-4)
Supplement: Supplementary file 2 — Supplementary Material 2. [file 12913_2024_11820_MOESM2_ESM.docx]

**Interview guide to reflect on the process of starting up the MiniMaria reception.**

Now the planning with Mini-Maria in Sandviken is in full swing. There is a steering group, a project group, and eventually a working group. If I understand correctly, you are part of the X group. I’d like to look back at when you heard about the MiniMaria project for the first time.

- Can you describe how and when you first heard about the Mini-Maria project in Sandviken?
- What were your initial thoughts and feelings when you learned about the project?
  - Did they change over time?
- When and how were you invited to participate in the X-group?
  - Was it an easy decision for you to accept?
- How did you come to that decision?
- How do you see your role in the project and how has it evolved since you started participating?
  - Have you taken on new responsibilities or tasks?
- What expectations do you think the other participants had on you in the project, both initially and as it progressed?
  - How do you manage these expectations?
- Can you describe any significant events or decisions that have influenced the project's development since you became involved?
- How did your previous experiences and skills help you contribute to the project's success?
- What challenges did you encounter during the project, and how did you and the group handle them?
- Are there any specific memories or achievements that stand out to you when you think about your time in the project so far?
- If you were to give advice to yourself at the beginning of the project, considering what you know now, what would it be?
- What expectations do you have for the project moving forward?
